# Genome-Wide Identification and Expression Analysis of the Class III Peroxidase Gene Family under Abiotic Stresses in Litchi (*Litchi chinensis* Sonn.)

**DOI:** 10.3390/ijms25115804

**Published:** 2024-05-26

**Authors:** Jie Yang, Rong Chen, Xu Xiang, Wei Liu, Chao Fan

**Affiliations:** Guangdong Provincial Key Laboratory of Tropical and Subtropical Fruit Tree Research, Key Laboratory of South Subtropical Fruit Biology and Genetic Resource Utilization, Ministry of Agriculture and Rural Affairs, Institute of Fruit Tree Research, Guangdong Academy of Agricultural Sciences, Guangzhou 510640, China; yangjie95222@126.com (J.Y.); rongchen9821@126.com (R.C.); xiangxu@vip.163.com (X.X.); liuwei1@gdaas.cn (W.L.)

**Keywords:** litchi, PRX gene family, phylogenetic tree, abiotic stresses, expression pattern

## Abstract

Class III peroxidases (CIII PRXs) are plant-specific enzymes with high activity that play key roles in the catalysis of oxidation-reduction reactions. In plants, CIII PRXs can reduce hydrogen peroxide to catalyze oxidation–reduction reactions, thereby affecting plant growth, development, and stress responses. To date, no systematic analysis of the CIII PRX gene family in litchi (*Litchi chinensis* Sonn.) has been documented, although the genome has been reported. In this study, a total of 77 CIII PRX (designated LcPRX) gene family members were predicted in the litchi genome to provide a reference for candidate genes in the responses to abiotic stresses during litchi growth and development. All of these *LcPRX* genes had different numbers of highly conserved PRX domains and were unevenly distributed across fifteen chromosomes. They were further clustered into eight clades using a phylogenetic tree, and almost every clade had its own unique gene structure and motif distribution. Collinearity analysis confirmed that there were eleven pairs of duplicate genes among the LcPRX members, and segmental duplication (SD) was the main driving force behind the *LcPRX* gene expansion. Tissue-specific expression profiles indicated that the expression levels of all the LcPRX family members in different tissues of the litchi tree were significantly divergent. After different abiotic stress treatments, quantitative real-time PCR (qRT-PCR) analysis revealed that the *LcPRX* genes responded to various stresses and displayed differential expression patterns. Physicochemical properties, transmembrane domains, subcellular localization, secondary structures, and cis-acting elements were also analyzed. These findings provide insights into the characteristics of the LcPRX gene family and give valuable information for further elucidating its molecular function and then enhancing abiotic stress tolerance in litchi through molecular breeding.

## 1. Introduction

Litchi (*Litchi chinensis* Sonn.) is a subtropical and tropical evergreen tree. It is a member of the Sapindaceae family and is a highly valuable fruit crop [1]. It originated in southern China, where its cultivation and propagation began during the Han Dynasty, and has a history spanning over two thousand years [2]. Litchi fruit stands out for its red color, unique flavor, good taste, rich nutrition, health effects, and increasing commercial value [3,4]. Furthermore, the litchi tree is popular and attractive because of its solid structure and corrosion resistance [5]. However, its growth and fruit yield are limited by adverse weather conditions, poor growing environments, and fruit tree pests and diseases [6]. It is very necessary for litchi growth and development to analyze the molecular mechanisms of resistance under multiple stresses.

Peroxidases (PRXs) are a type of antioxidant enzyme that are widely present in organisms, including animals, plants, and microorganisms [7]. As we know, PRXs can catalyze oxidation–reduction reactions by moving hydrogen peroxide (H_2_O_2_) to various organic and inorganic compounds [8]. More narrowly, PRXs can effectively remove excessive free radicals inside the organism that are stress-induced, protect cells from oxidative damage, and relieve the toxic effect of H_2_O_2_ [9]. And research has also demonstrated that PRXs can enhance the activity of natural killer (NK) cells [10], regulate cell proliferation, differentiation, and apoptosis [11], and protect sensitive free radical proteins [12]. According to their protein sequences and structural characteristics, PRXs are divided into two categories, including heme PRXs and non-heme PRXs [13]. Heme PRXs are further divided into animal PRXs and non-animal PRXs [14]. Yet non-animal PRXs are divided into three major classes, namely, Class I Peroxidases (CI PRXs), Class II Peroxidases (CII PRXs), and Class III Peroxidases (CIII PRXs) [15]. These three classes of PRXs have similar three-dimensional structures, low homologous amino acid sequences, and different reaction mechanisms [16]. CI PRXs, which are intracellular enzymes that are widely distributed in living organisms except animals, including plant ascorbate PRXs, yeast cytochrome c PRX, and bacterial catalase-PRXs, remove excess H_2_O_2_ to prevent cell damage [17,18]; CII PRXs are exclusively extracellular fungal enzymes that play a vital role in the process of degrading lignins [19]; CIII PRXs, also known as typical secretory plant PRXs, are a large multigene family in various plants and are commonly secreted into the cell walls or the cell-wall constituents or the vacuoles [20,21].

CIII PRXs are plant-specific glycoproteins that are involved in glycosylation by binding to carbohydrate side chains [22]. CIII PRXs have been shown to participate in many vital physiological processes in plants, including cell wall formation [23], wound healing [24], auxin metabolism [25,26], lignification [27], and stress responses [28]. Until now, CIII PRXs have been reported from Arabidopsis (*Arabidopsis thaliana*) [29], rice (*Oryza sativa*) [30], Chinese pear (*Pyrus bretschneideri*) [31], cassava (*Manihot esculenta*) [32], sweet orange (*Citrus Sinensis*) [33], potato (*Solanum tuberosum*) [34], and soybean (*Glycine max*) [35]. There is growing evidence that CIII PRXs are abiotic stress-responsive enzymes in different plant species; for example, *AtPRX3* (*ATRCI3*) could positively regulate drought and salt tolerance in Arabidopsis [36]; *AtPRX22*/*39*/*69* could positively regulate freezing tolerance in Arabidopsis [37]; overexpression of *AtPRX64* could promote root growth, reduce reactive active oxygen and aluminum accumulation in the roots, and improve the tolerance of tobacco (*Nicotiana tabacum*) to aluminum stress [38]; overexpression of *OsPRX38* in Arabidopsis enhanced tolerance of arsenic stress in transgenic line [39]; *CrPRX*, a *Catharanthus roseus CIIIPRX* gene, enhanced tolerance of salt and drought stresses in transgenic tobacco [40]; the expression of *ZmPRX26*/*42*/*71*/*75*/*78* were predominantly observed in the roots of maize (*Zea mays*) and exhibited significant upregulation under drought and salt stresses [28]; *HbPRX42* could enhance tolerance of drought stress via multiple phytohormone signaling pathways in rubber tree (*Hevea brasiliensis*) [41]; *RsPRX1*, repressed by RNAi, enhanced salt, antioxidant and heat tolerance in radish (*Raphanus sativus*) [42]; *StPRX41*/*57* in potato have been shown to enhance plant susceptibility to drought stress, and their expression levels increased four times compared to the control [34]; overexpression of *TaPRX-2A* in wheat exhibited an enhanced tolerance to salt stress [43].

Although the identification and function of the CIII PRX gene family have been extensively studied in various plants, no reports on litchi are available. The present study is the first to report on a systematic investigation of the CIII PRX (designated *LcPRX*) genes in litchi based on a recently published genome database [1] and large-scale transcriptome data. In the current study, we searched for all nonredundant sets of *LcPRX* genes and performed their analysis, including chromosome location, phylogenetic relationship, gene duplication, gene structures, and cis-acting elements. Moreover, the expression patterns of 77 *LcPRX* genes in different tissues of litchi were also analyzed. Furthermore, we carried out *LcPRX* gene expression analysis under various abiotic stress treatments (cold, heat, drought, and salt) using quantitative real-time PCR (qRT-PCR). The obtained results distinctly represent an exceptionally useful resource for future in-depth studies on the evolution, structures, expression, and functions of LcPRX family members, which will assuredly be very helpful in the future exploration of stress-related responses in litchi. Undoubtedly, all of these will provide a theoretical basis and genetic resources for cultivating varieties with strong stress resistance, enabling targeted genetic improvement to be carried out to improve the survival and production capacities of litchi.

## 2. Results

### 2.1. Genome-Wide Identification and Evolution of LcPRX Genes

In the present study, 77 putative *PRX* genes were confirmed in the litchi genome using the HMMER 3.0 software and conservative domain identification. Then, they were designated as *LcPRX1-LcPRX77* according to the order of their position on the fifteen chromosomes, starting with the first on chromosome 1 labeled *LcPRX1*. Their physical and chemical properties are presented in Table S3. The coding sequence (CDS) length of the 77 *LcPRX* genes ranged from 729 bp (*LcPRX64*) to 1710 bp (*LcPRX6*), and they contained between 242 amino acids (aa) (*LcPRX64*) and 569 aa (*LcPRX6*). The significant variation in molecular mass spanned from 26.05 kDa (*LcPRX64*) to 63.58 kDa (*LcPRX6*). The theoretical isoelectric point (pI) fluctuated between 4.38 (*LcPRX18*) and 9.71 (*LcPRX48*), signifying predominantly weakly basic proteins. Accordingly, the instability index varied from 26.14 (*LcPRX23*) to 55.66 (*LcPRX20*), signifying predominantly stable proteins. Moreover, except for *LcPRX1*/*7*/*18*/*27*/*37*/*56*/*58*/*59*/*62*/*66*/*68*/*71*, the grand average of hydropathicity (GRAVY) values in the remaining 65 *LcPRX* genes was less than zero, signifying predominantly hydrophilic proteins. Furthermore, the number of transmembrane domains in 77 LcPRX proteins ranged from 0 to 2 (*LcPRX7*/*71*). On the other hand, the subcellular localization was very diverse, with 41 (*LcPRX1*/*2*/*10*/*11*/*12*/*14*/*15*/*16*/*17*/*19*/*21*/*22*/*23*/*24*/*25*/*28*/*32*/*33*/*36*/*37*/*38*/*40*/*42*/*43*/*48*/*50*/*52*/*54*/*57*/*58*/*61*/*62*/*63*/*64*/*66*/*69*/*71*/*72*/*73*/*74*/*77*) being assigned to the chloroplast, 20 (*LcPRX3*/*5*/*8*/*18*/*20*/*27*/*30*/*31*/*34*/*35*/*41*/*44*/*47*/*51*/*53*/*55*/*59*/*60*/*68*/*76*) as extracellular, 6 (*LcPRX9*/*29*/*39*/*49*/*67*/*75*) as cytoplasm, 5 (*LcPRX4*/*7*/*26*/*46*/*56*) as vacuole, 4 (*LcPRX6*/*13*/*45*/*70*) as nucleus, and 1 (*LcPRX65*) as plasma membrane.

**Figure 1:**
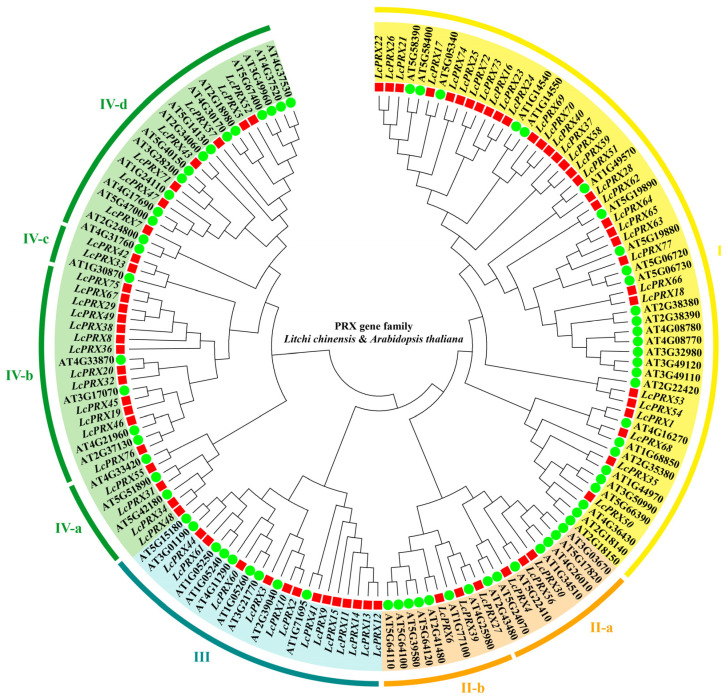
Phylogenetic tree of PRX proteins from litchi and Arabidopsis. Red squares represent *LcPRX* genes, and green circles represent *AtPRX* genes. Clusters (I–IV) are displayed in different colors. Clusters are divided into different subfamilies (a–d) according to their evolutionary distance.

To define the evolutionary relationships and the basic classifications among the *PRX* genes in litchi, a phylogenetic tree was constructed by aligning 77 LcPRX protein sequences and 73 AtPRX protein sequences based on a dicotyledonous model plant (Arabidopsis) reference. According to this phylogenetic tree (Figure 1), all PRX proteins were clustered into eight subfamilies. Subfamily I contained 32 LcPRX proteins (*LcPRX1*/*16*/*17*/*18*/*21*/*22*/*23*/*24*/*25*/*26*/*28*/*35*/*37*/*40*/*50*/*51*/*53*/*54*/*58*/*59*/*62*/*63*/*64*/*65*/*66*/*68*/*69*/*70*/*72*/*73*/*74*/*77*), subfamily II-a contained 3 (*LcPRX4*/*30*/*56*), subfamily II-b contained 3 (*LcPRX6*/*27*/*39*), subfamily III contained 13 (*LcPRX2*/*3*/*9*/*10*/*11*/*12*/*13*/*14*/*15*/*41*/*44*/*60*/*61*), subfamily IV-a contained 4 (*LcPRX31*/*34*/*48*/*55*), subfamily IV-b contained 13 (*LcPRX8*/*19*/*20*/*29*/*32*/*36*/*38*/*45*/*46*/*49*/*67*/*75*/*76*), subfamily IV-c contained 2 (*LcPRX33*/*42*), and the remaining 7 (*LcPRX5*/*7*/*43*/*47*/*52*/*57*/*71*) belonged to subfamily V. The results showed that the evolution of *LcPRX* genes was consistent with that of *AtPRX* genes, proving that PRX proteins are relatively conserved in evolution.

### 2.2. Phylogenetic Relationship, Gene Structure, and Motif Composition of LcPRX Genes

**Figure 2:**
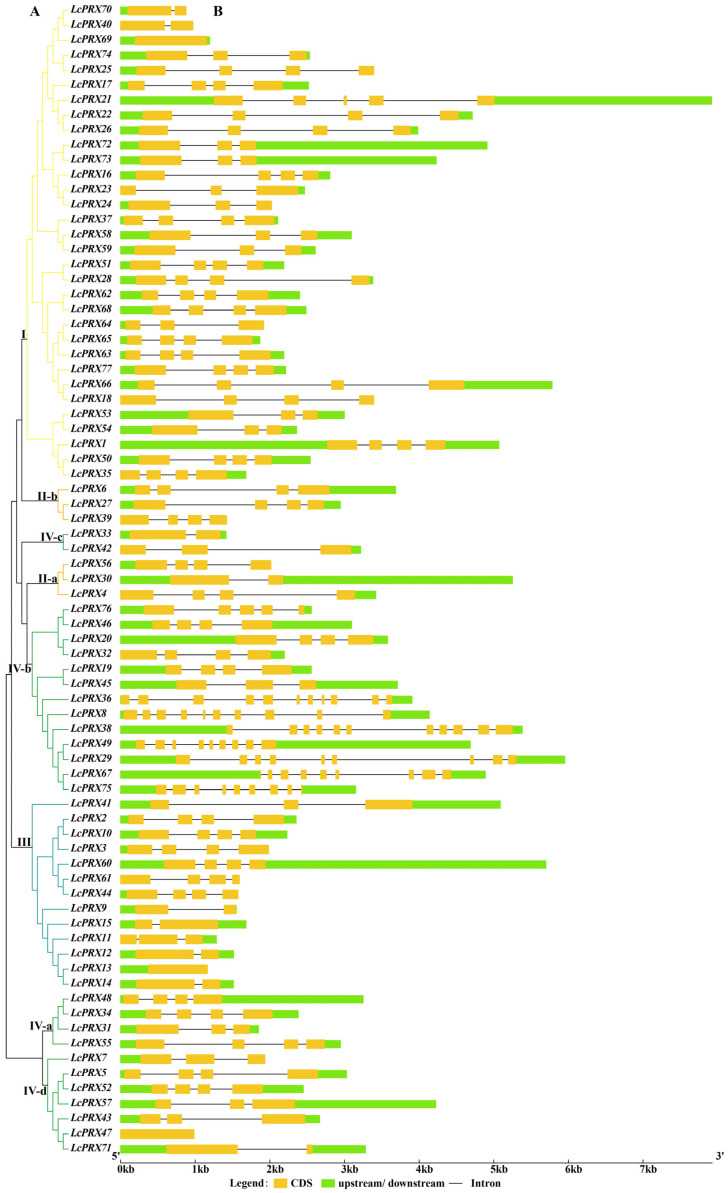
Phylogenetic relationship and gene structure analysis of *LcPRX* genes. (**A**) Phylogenetic tree of 77 *LcPRX* proteins. (**B**) Exon–intron structure of *LcPRX* genes. Yellow boxes indicate exons, lines indicate introns, and green boxes indicate upstream or downstream.

As show in Figure 2A, the 77 *LcPRX* genes were divided into eight subfamilies, aligning with the unrooted phylogenetic tree (Figure 1). To better understand the evolutionary conservation of this gene family, we elucidated the gene structures. The results indicated that the *LcPRX* genes had relatively complex gene structures, as the intron number spanned from zero to ten (Figure 2B). Three intron-free *LcPRX* genes (*LcPRX13*/*47*/*69*) were predicted, including one gene belonging to subfamily III, one belonging to IV-d, and one belonging to I. Two genes (*LcPRX36*/*38*) with ten introns were predicted, and they all belonged to subfamily IV-b. *LcPRX* genes with closer relationships exhibited obvious structural conservation; for example, all members of subfamily II-b had four exons and three introns. It was worth noting that *LcPRX31*, which was quite different from other IV-a genes, had three exons and two introns, showing that this gene might have a different evolutionary pattern.

**Figure 3:**
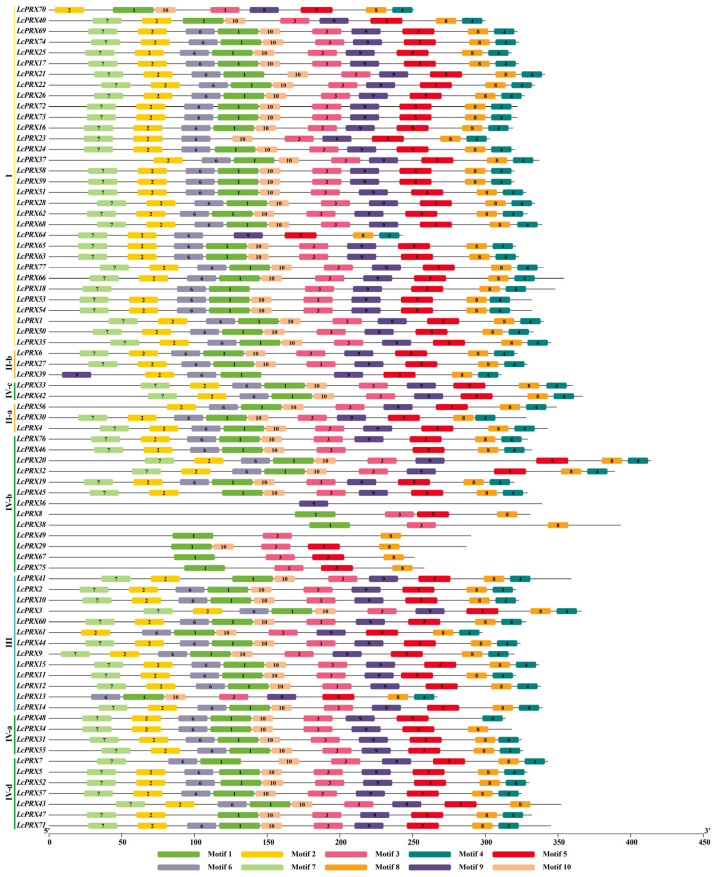
Conserved motif analysis of *LcPRX* genes. Ten putative motifs are indicated in different colored boxes. Detailed conserved motif information is listed in Table S4.

To further infer the potential diversified functions of *LcPRX* genes, we detected the conserved motifs. We found 10 conserved motifs were established (Figure 3), whose amino acid sequences are shown in Table S4. Notably, the 77 *LcPRX* genes had a varying number of PRX domains (Motif 1/2/3/4/5/6/7/8/9/10). 1 gene (*LcPRX36*) had the fewest amino acid sequences with one motif (Motif 9), while 52 genes (*LcPRX1*/*2*/*3*/*4*/*5*/*6*/*9*/*10*/*11*/*12*/*14*/*15*/*16*/*17*/*19*/*20*/*21*/*22*/*24*/*25*/*26*/*27*/*28*/*30*/*31*/*32*/*33*/*35*/*42*/*44*/*50*/*51*/*52*/*53*/*54*/*55*/*57*/*58*/*59*/*60*/*62*/*63*/*65*/*66*/*68*/*69*/*71*/*72*/*73*/*74*/*76*/*77*) had the most with ten (Motif 1/2/3/4/5/6/7/8/9/10), other genes contained motifs with the number ranging from three (*LcPRX38*/*49*/*67*) to nine (*LcPRX7*/*23*/*34*/*37*/*40*/*41*/*43*/*45*/*46*/*47*/*48*/*56*/*61*). *LcPRX* genes that were relatively close to each other had similar motif distribution; for example, *LcPRX5*/*52*/*57*/*71* both had ten motifs and had no significant differences in type and length. Moreover, the motif composition of *LcPRX39* was quite unique, having two Motif 9s, indicating that the protein sequences of *LcPRX39* had undergone tandem repeat (TR) in Motif 9.

### 2.3. Chromosomal Distribution and Duplication Events of LcPRX Genes

**Figure 4:**
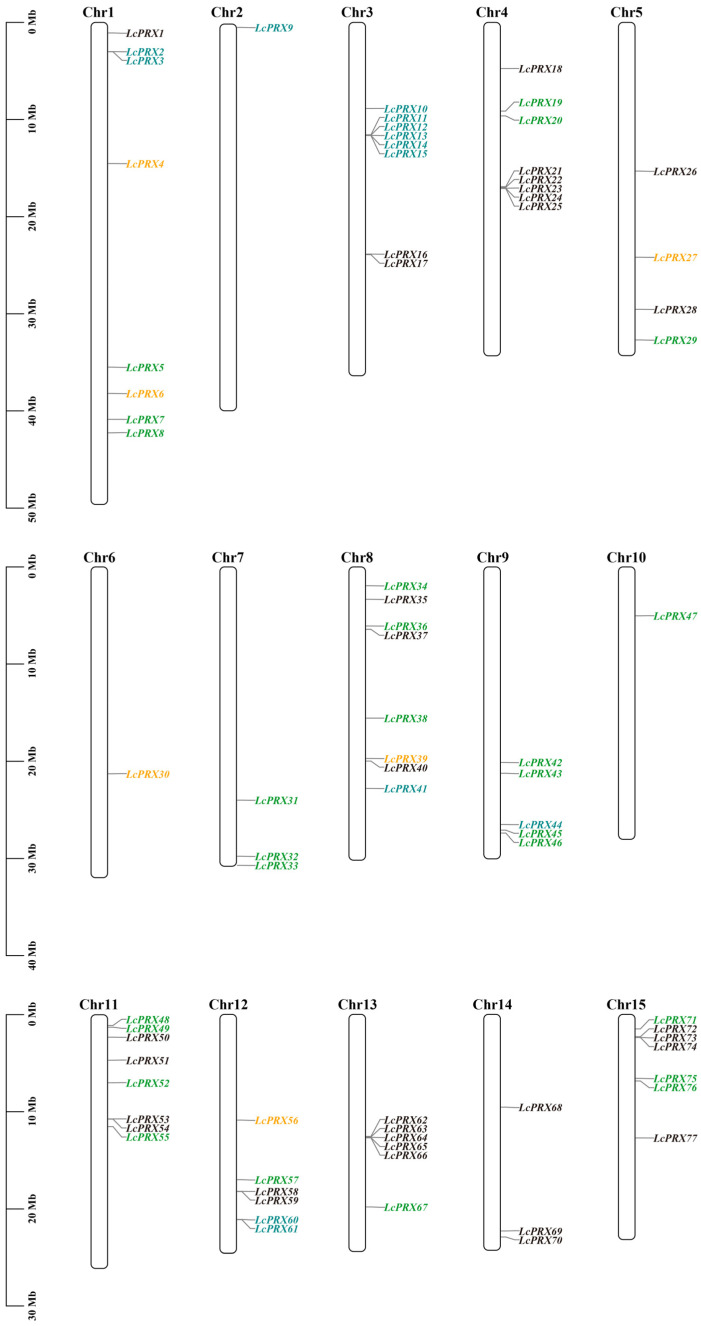
Chromosomal location of *LcPRX* genes. The bars represent chromosomes. The chromosome numbers are displayed on the top side, and the gene names are displayed on the right side. Different colors represent different subfamilies. Detailed chromosomal location information is listed in Table S5.

In this study, 77 *LcPRX* genes were unevenly distributed among fifteen chromosomes based on their positions in the litchi genome (Figure 4 and Table S5). Among them, *LcPRX* genes were mainly seen on the first, third, fourth, eighth, eleventh, thirteenth, and fifteenth chromosomes, and the number of *LcPRX* genes varied from 1 on the second chromosome to 9 on the fourth chromosome. Obviously, the chromosome length was not positively correlated with the number of *LcPRX* genes contained on the chromosome. In addition, different *LcPRX* genes contained on the same chromosome were usually classified as different subfamilies in the phylogenetic relationship within litchi, suggesting that different *LcPRX* genes contained on a chromosome might perform different functions.

**Figure 5:**
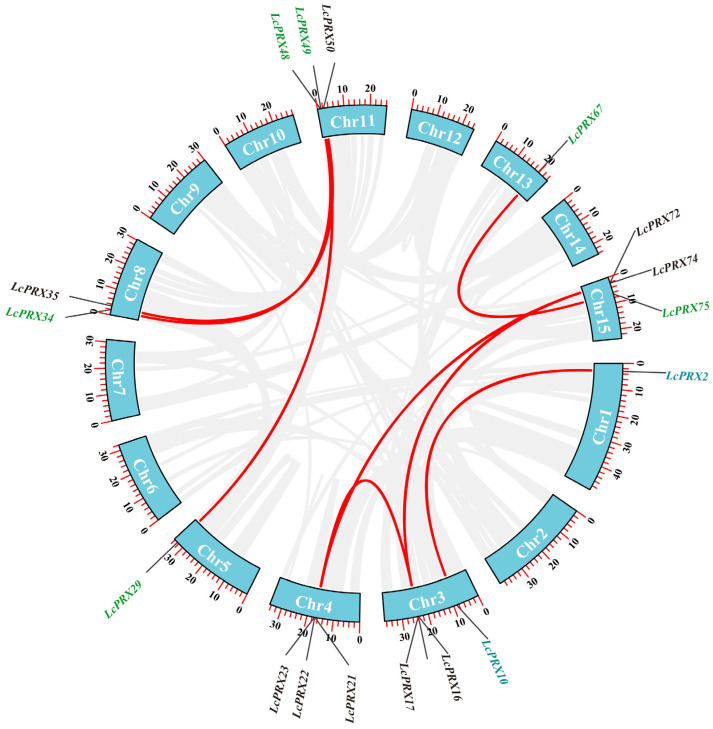
Duplication events of the *LcPRX* genes. Different gene colors represent the different subfamilies. Red curves linking *LcPRX* genes indicate duplicated gene pairs, and the gray curves indicate duplicated gene pairs in litchi.

To understand the expansions of *LcPRX* genes, we identified the gene duplication events. Combined with the distribution results, there were no pairs of tandem duplication (TD) events, and there were eleven pairs of segmental duplication (SD) events in duplicated *LcPRX* genes (Figure 5 and Table S6). This indicated that SD was the main driving force behind the expansion of *LcPRX* genes. For all duplicated gene pairs, the ratios of non-synonymous (Ka) to synonymous (Ks) were below one (Table S7), which generally indicates purifying selection [44]. Therefore, purifying selection was the major reason for the increase in *LcPRX* gene numbers.

**Figure 6:**
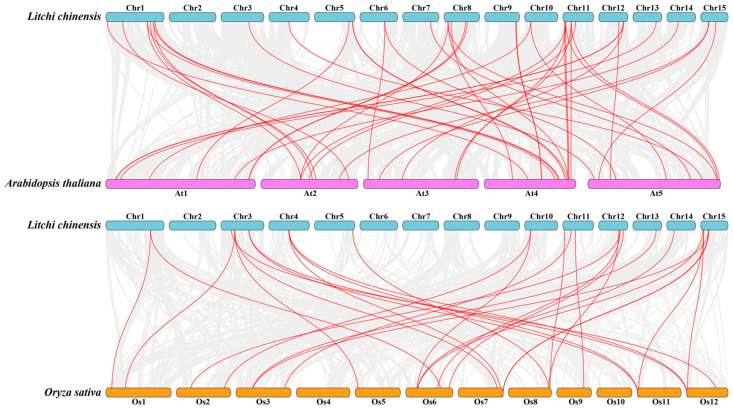
The collinearity relationship between litchi and other species. The collinear relationships between the litchi, Arabidopsis, and rice genomes are shown on the chromosome maps. The gray lines represent the collinearity between all genes, and the red lines represent the collinearity between *PRX* genes.

To better study the evolutionary relationships of *PRX* genes between various species, the syntenic relationships were analyzed by constructing two intergenomic collinear maps with Arabidopsis and rice. There were forty-three and twenty-eight pairs of orthologous genes between litchi, Arabidopsis, and rice, respectively (Figure 6, Tables S8 and S9). The results indicated that, compared to rice, litchi and Arabidopsis had a closer relationship.

### 2.4. Secondary Structure and Promoter Region Analysis of LcPRX Genes

The secondary structure analysis revealed that the 77 LcPRX proteins all had an alpha helix, an extended strand, a beta turn, and a random coil (Table S10). Among these structures, a random coil accounted for the largest proportion in 50 of the LcPRX proteins, while an alpha helix had the largest share in the rest of the LcPRX proteins. In particular, random coil > alpha helix > extended strand > beta turn was analyzed in 49 (*LcPRX1*/*3*/*4*/*6*/*7*/*8*/*9*/*11*/*13*/*15*/*18*/*19*/*20*/*21*/*23*/*26*/*28*/*29*/*30*/*31*/*32*/*33*/*35*/*37*/*38*/*39*/*42*/*43*/*45*/*47*/*48*/*49*/*51*/*53*/*54*/*55*/*57*/*59*/*60*/*61*/*63*/*64*/*65*/*66*/*67*/*68*/*70*/*74*/*77*) of the 77 LcPRX proteins, followed by random coil = alpha helix > extended strand > beta turn in 1 (*LcPRX16*) of the remaining 28 LcPRX proteins, and alpha helix > random coil > extended strand > beta turn in the remaining LcPRX proteins (*LcPRX2*/*5*/*10*/*12*/*14*/*17*/*22*/*24*/*25*/*27*/*34*/*36*/*40*/*41*/*44*/*46*/*50*/*52*/*56*/*58*/*62*/*69*/*71*/*72*/*73*/*75*/*76*). The results indicated that LcPRX proteins had complex structures and reflected their functional diversity.

**Figure 7:**
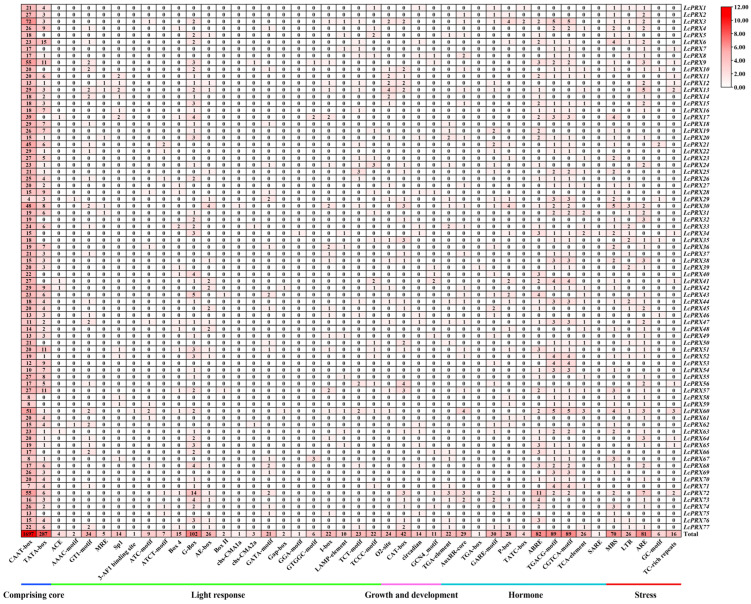
Cis-acting elements of *LcPRX* genes. Numbers of different elements in the promoter region of *LcPRX* genes, as indicated by different color intensities and numbers in the grid.

To gain insight into the transcriptional regulation and potential functions of *LcPRX* genes, we predicted the cis-acting elements. A total of 44 different types of the 3007 cis-acting elements were calculated (Figure 7 and Table S11). These elements were divided into five categories, comprising core elements (CAAT-box, TATA-box), light response elements (ACE, AAAC-motif, GT1-motif, MRE, Sp1, 3-AF1 binding site, ATC-motif, ATCT-motif, Box 4, G-Box, AE-box, Box II, chs-CMA1a, chs-CMA2a, GATA-motif, Gap-box, GGA-motif, GTGGC-motif, I-box, LAMP-element, TCT-motif, and TCCC-motif), growth and development response elements (O2-site, CAT-box, circadian, and GCN4_motif), hormone response elements (TGA-element, AuxRR-core, TGA-box, GARE-motif, P-box, TATC-box, ABRE, TGACG-motif, CGTCA-motif, TCA-element, and SARE), and stress response elements (MBS, LTR, ARE, GC-motif, and TC-rich repeats). Specifically, the number of core elements was 1984. Similarly, the most common elements were light response elements, which distributed unevenly on all *LcPRX* genes. The numbers of elements related to light responsiveness and part of a light response were 60 and 272, respectively. Meanwhile, growth and development response elements were predicted to reach four types; the numbers of elements related to zein metabolism regulation, meristem expression, circadian control, and endosperm expression were 24, 42, 14, and 11, respectively. Moreover, hormone response elements were predicted to reach five types; the numbers of elements related to auxin, gibberellin, abscisic acid, methyl jasmonic, and salicylic acid were 52, 62, 82, 178, and 27, respectively. Furthermore, stress response elements were predicted to reach five types; the numbers of elements related to drought, low temperature, anaerobic induction, anoxic induction, and defense and stress were 70, 26, 81, 6, and 16, respectively. These data suggested that *LcPRX* genes were responsive to various factors, including light, growth and development, hormones, and stress, and these response elements may directly participate in the expression and regulation of *LcPRX* genes during growth and development as well as different stress conditions.

### 2.5. Expression Patterns of LcPRX Genes in Different Tissues

**Figure 8:**
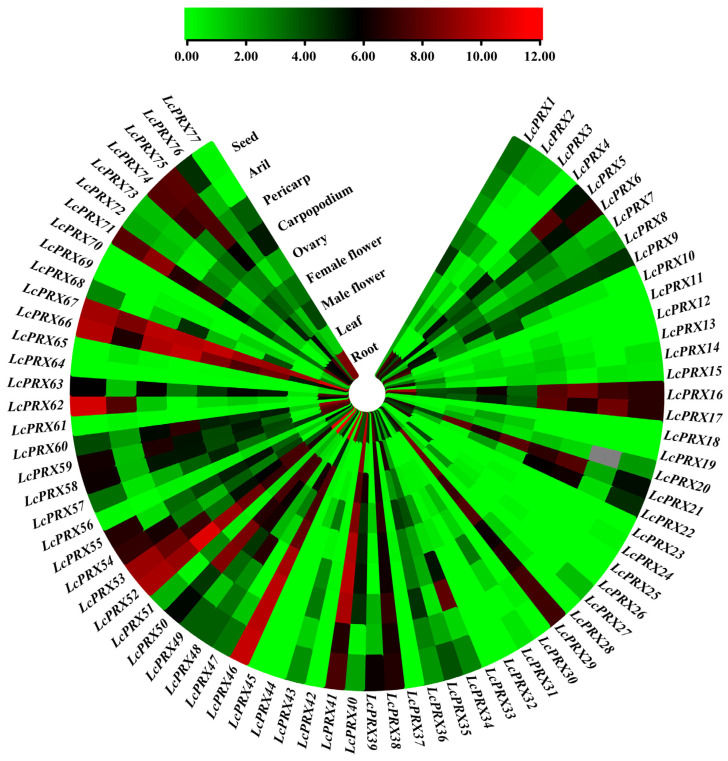
Expression patterns of *LcPRX* genes in different tissues. Red represents induced expression, and green represents repressed expression.

The RNA-seq results in Figure 8 show the expression patterns of 77 *LcPRX* genes across nine distinct tissues, and their details are listed in Table S12. We found from the results that the 77 *LcPRX* genes had different tissue-specific expression patterns, with expression observed in at least one of the nine tissues. All expressed genes could be broadly classified into four categories based on their expression patterns, including the following: the category A genes exhibited only in one tissue and were not present in others: *LcPRX11*/*18*/*23*/*64*/*70* were exclusively expressed in roots, and *LcPRX25* in female flowers; the category B genes were only expressed in a few (with less than five) tissues and were not present in others: *LcPRX4*/*28*/*33* exhibited only in four of the nine tissues, *LcPRX19*/*24*/*26*/*27*/*37* exhibited only in three of the nine tissues, and *LcPRX30*/*32*/*42*/*44*/*45*/*61* exhibited only in two of the nine tissues; the category C genes were expressed in multiple (with more than four) tissues: *LcPRX2*/*10*/*15*/*31*/*62*/*68* exhibited in seven tissues, *LcPRX3*/*13*/*56* exhibited in six tissues, *LcPRX5*/*14*/*20*/*34*/*43*/*54*/*59*/*72*/*73*/*77* exhibited in eight tissues, and *LcPRX12*/*39*/*47*/*65*/*69* exhibited in five tissues; the category D genes were expressed in all nine tissues: these were the remaining 33 *LcPRX* genes. Moreover, except for individual genes, gene expression patterns in the same subfamily exhibited similarity; for example, subfamily IV-a, IV-b, and IV-d genes showed more or less expression in almost all tissues, suggesting that they might be extensively involved in the growth and development of litchi; subfamily II-a, II-b, and IV-c genes were expressed only in a few individual tissues, suggesting that they were only involved in the growth and development of one part of litchi. Overall, these results showed that the 77 *LcPRX* genes exhibited varied expression in different tissues, which may be responsible for the functional diversity of *LcPRX* genes.

### 2.6. Expression Analysis of LcPRX Genes after Stress Treatments

**Figure 9:**
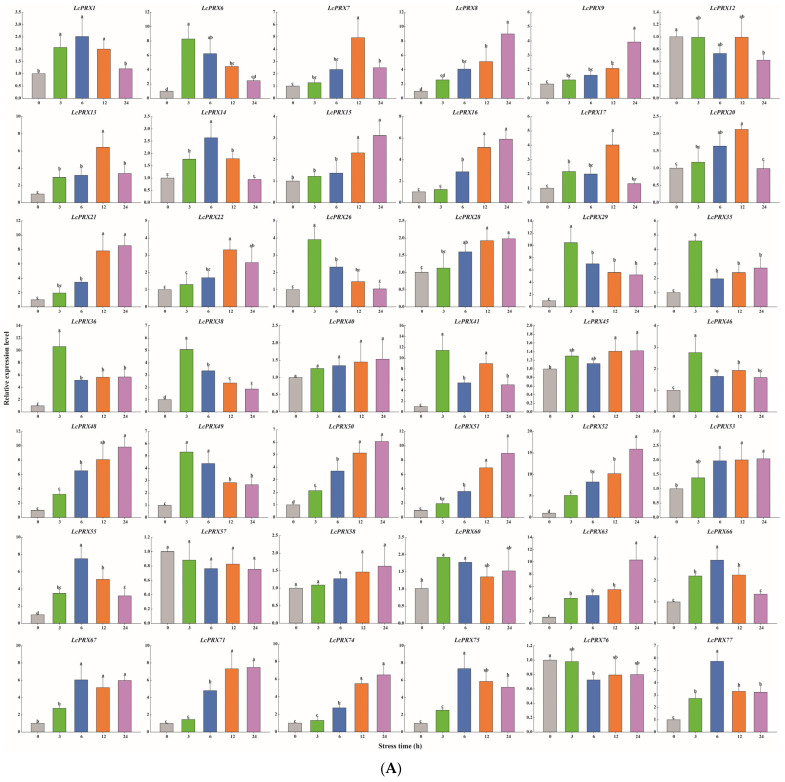

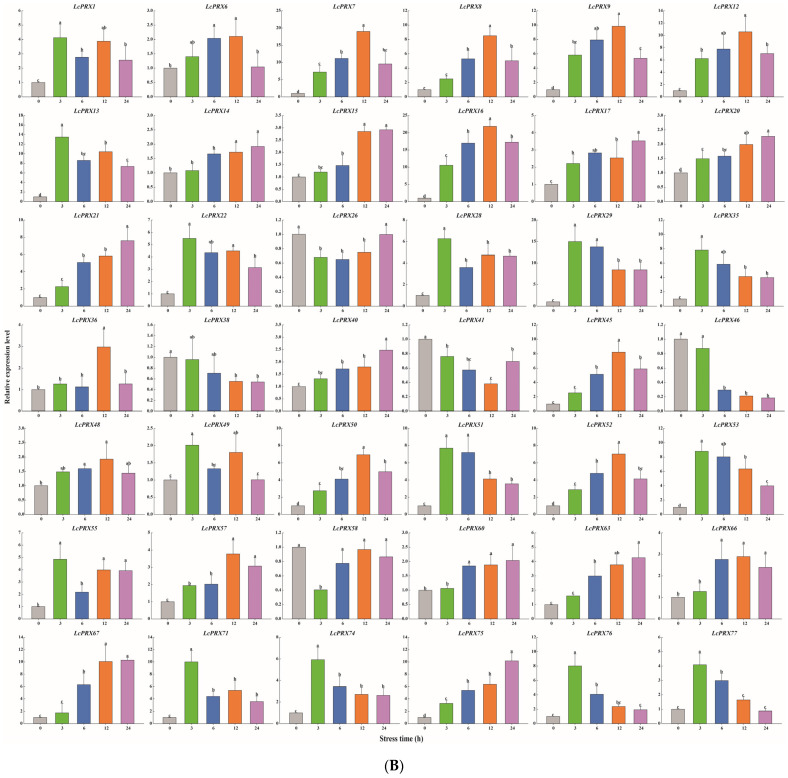

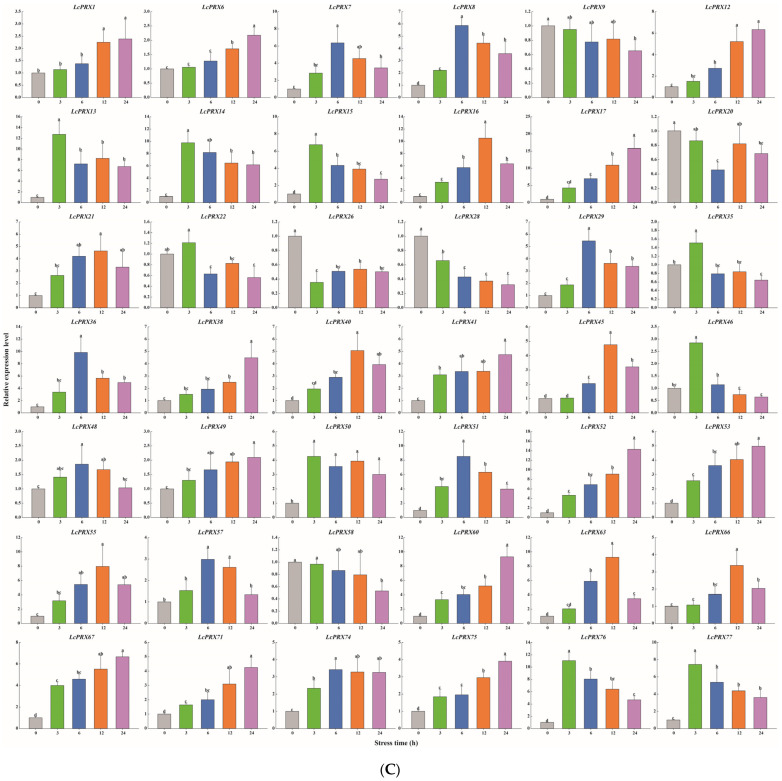

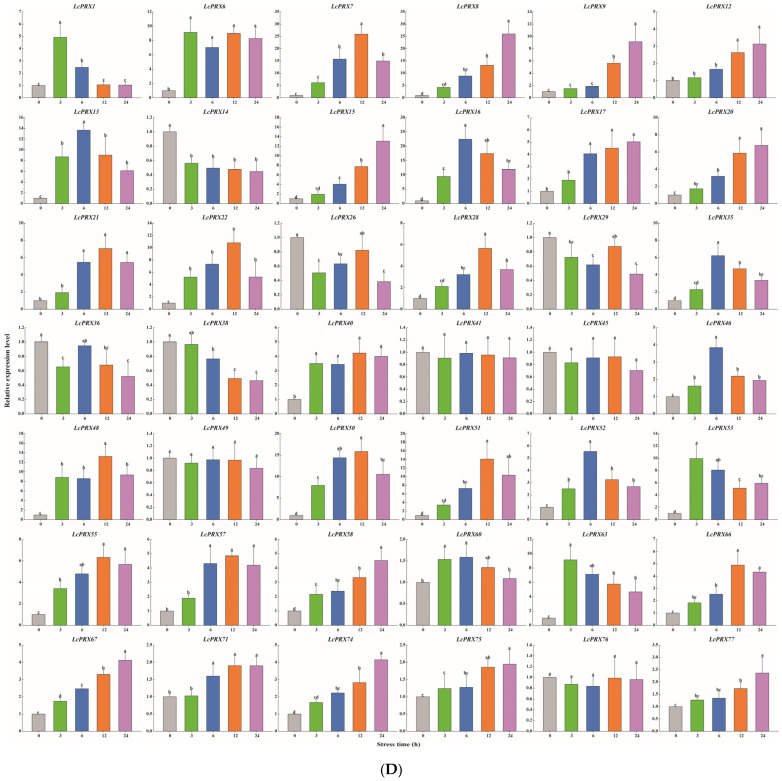
Relative expression of *LcPRX* genes after stress treatments. (**A**) cold (4.0 ± 1.0 °C), (**B**) heat (38.0 ± 0.5 °C), (**C**) drought (20% (*w*/*v*) PEG6000), and (**D**) salt (400 mmol/L NaCl). Error bars represent the standard deviation of three replicates. Different lowercase letters indicate significant differences (*p* < 0.05).

On the basis of the cis-acting elements data and the RNA-seq data (which combined the nine tissues of litchi), we picked 42 *LcPRX* genes (*LcPRX1*/*6*/*7*/*8*/*9*/*12*/*13*/*14*/*15*/*16*/*17*/*20*/*21*/*22*/*26*/*28*/*29*/*35*/*36*/*38*/*40*/*41*/*45*/*46*/*48*/*49*/*50*/*51*/*52*/*53*/*55*/*57*/*58*/*60*/*63*/*66*/*67*/*71*/*74*/*75*/*76*/*77*) that were expressed in leaves and responded to hormones and stresses to detect their expression levels after stress treatments (cold, heat, drought, and salt). Altogether, all of these 42 genes displayed differential expression compared to the untreated control (0 h) after at least one stress treatment (Figure 9).

After cold treatment, the expression levels of 10 *LcPRX* genes (*LcPRX6*/*26*/*29*/*35*/*36*/*38*/*41*/*46*/*49*/*60*) came up to the top at 3 h, indicating that these genes could quickly respond to cold stress; similarly, 12 genes (*LcPRX1*/*7*/*13*/*14*/*17*/*20*/*22*/*55*/*66*/*67*/*75*/*77*) had the highest expression levels at 6 h or 12 h; 17 genes (*LcPRX8*/*9*/*15*/*16*/*21*/*28*/*40*/*45*/*48*/*50*/*51*/*52*/*53*/*58*/*63*/*71*/*74*) were continuously up-regulated from 0 h to 24 h, proving that these genes might not have completed their response to cold stress; 3 genes (*LcPRX12*/*57*/*76*) were down-regulated compared to 0 h after other arbitrary time points. After heat treatment, 14 genes (*LcPRX1*/*13*/*22*/*28*/*29*/*35*/*49*/*51*/*53*/*55*/*71*/*74*/*76*/*77*) were rapidly up-regulated at 3 h; 13 genes (*LcPRX6*/*7*/*8*/*9*/*12*/*16*/*36*/*45*/*48*/*50*/*52*/*57*/*66*) reached the highest expression levels at 6 h or 12 h, while 10 genes (*LcPRX14*/*15*/*17*/*20*/*21*/*40*/*60*/*63*/*67*/*75*) were up-regulated to the highest levels at 24 h; the expression levels of 5 genes (*LcPRX26*/*38*/*41*/*46*/*58*) decreased compared to 0 h at any other time points. After drought treatment, the expression levels of 9 genes (*LcPRX13*/*14*/*15*/*22*/*35*/*46*/*50*/*76*/*77*) sharply increased at 3 h, followed by trends of down-regulation from 3 h to 24 h; the increasing trends of 15 genes (*LcPRX7*/*8*/*16*/*21*/*29*/*36*/*40*/*45*/*48*/*51*/*55*/*57*/*63*/*66*/*74*) expression were the most obvious at 6 h or 12 h; 13 genes (*LcPRX1*/*6*/*12*/*17*/*38*/*41*/*49*/*52*/*53*/*60*/*67*/*71*/*75*) showed maximum responsiveness at 24 h; 5 genes (*LcPRX9*/*20*/*26*/*28*/*58*) were inhibited at other time points compared to 0 h. After salt treatment, 4 genes (*LcPRX1*/*6*/*53*/*63*) exhibited the highest trends from 0 h to 3 h; the expression levels of 18 genes (*LcPRX7*/*13*/*16*/*21*/*22*/*28*/*35*/*40*/*46*/*48*/*50*/*51*/*52*/*55*/*57*/*60*/*66*/*71*) were highest at 6 h or 12 h; 11 genes (*LcPRX8*/*9*/*12*/*15*/*17*/*20*/*58*/*67*/*74*/*75*/*77*) showed an upward expression trend between 0 h and 24 h; 9 genes (*LcPRX14*/*26*/*29*/*36*/*38*/*41*/*45*/*49*/*76*) exhibited low expression levels after any of these time points (except for 0 h). In summary, all of these 42 *LcPRX* genes had undergone complex and diverse responses to cold, heat, drought, and salt, indicating their indispensable regulatory role in adapting to the abiotic stresses of litchi.

## 3. Discussion

CIII PRXs are a large multigene family in plants and have key roles in the responses to unfavorable natural conditions during plant growth and development [45]. While systematic and comprehensive genome-wide analyses of PRX gene families in Arabidopsis [29], rice [30], and sweet orange [33] have been reported, a systematic genome-wide identification of the PRX gene family has yet to be studied in litchi. A high-quality litchi genome sequence [1] provides a foundation for the identification and characterization of the LcPRX gene family at the whole-genome level. Here, 77 putative *LcPRX* genes from the litchi genome were identified, and they were named from *LcPRX1* to *LcPRX77* according to their order on the chromosomes (Table S3). The number of *PRX* genes in litchi is similar to that in Arabidopsis (73) [29], sweet orange (72) [33], and carrot (*Daucus carota*) (75) [7], but significantly less than that in rice (138) [30], maize (119) [28], and *Brachypodium distachyon* (151) [46], which may be due to factors such as genome size [47] or genome duplication [48]. The molecular masses of PRX proteins usually range from 25 kDa to 65 kDa, such as those in Chinese cabbage (*Brassica rapa*) (27.39–47.67 kDa) [49], tobacco (*Nicotiana tabacum*) (27.45–54.50 kDa) [50], and foxtail millet (*Setaria italica*) (27.33–57.24 kDa) [51]. In the present study, the 77 LcPRX proteins had molecular masses of 26.05 kDa to 63.58 kDa, which were within the range of PRX proteins. Notably, the prediction of subcellular localization revealed that many LcPRX proteins (41) were located in the chloroplast, which is consistent with research reports on olive (*Olea europaea*) [52] and cabbage (*Brassica oleracea*) [53]. According to the above analysis, we speculate that PRX proteins function mainly in the chloroplasts of various plants. 

Structural diversity, especially alterations in exon–intron structure, is considered to be one of the primary driving forces behind the evolution of multigene families [34]. There are three main types in which exon–intron structure divergences were accomplished, namely exon/intron gain/loss, exonization/pseudo-exonization, and insertion/deletion [54]. Exon/intron gain/loss has been suggested to represent one of the main causes of gene family expansion in plants [28]. The results of the gene structure analysis showed that the 77 *LcPRX* genes had between zero and ten introns, while more than half of them (40) contained three or four introns (Figure 2). This is supported by the gene structure in Arabidopsis [29] and rice [30], proving that it is an ancestral structural model of *PRX* genes. Moreover, we have seen that most *LcPRX* genes showed similar structures and were grouped in the same subfamily. Similar phenomena have also been found in cassava [32] and sugarcane (*Saccharum*) [14]. Notably, however, a few genes (such as *LcPRX30*/*31*) in the same subfamily were not completely identical in terms of their intron/exon structure. This means that exon/intron loss may be a main pattern of LcPRX family evolution, which might be a great contributor to the functional diversity of *LcPRX* genes. Moreover, gene duplications play an important role in the evolution of genomes and genetic systems [55]. In this study, eleven pairs of duplicate *LcPRX* genes were found (Figure 5), and all these genes were involved in SD, implying that SD was the main contributor to the amplification of *LcPRX* genes. Furthermore, the Ka/Ks ratios of 11 pairs of genes showed that purifying selection might be principally responsible for maintaining the functions of *LcPRX* genes, this is similar to the report in Chinese pear [31].

It is known that the cis-acting elements exist in the genetic flanking sequences, which have the function of transcriptional regulation. More narrowly, as a kind of binding site, cis-acting elements regulate and control the expression of target genes by binding to transcription factors [56]. Many studies have shown that gibberellin, abscisic acid, methyl jasmonic, and salicylic acid can promote abiotic stress signal transduction, thereby enhancing the anti-adversity ability of plants [57,58,59,60]. These hormones affect their corresponding response elements (GARE-motif, P-box, TATC-box, ABRE, TGACG-motif, CGTCA-motif, TCA-element, and SARE) to regulate the expression of stress-related genes, which may result in the improvement of stress-resistant ability in unfavorable environments [61]. Moreover, diverse stress cis-elements (MBS, LTR, ARE, GC-motif, and TC-rich repeats) among genes also reflect their great potential for stress resistance [62]. In tobacco, it is speculated that the resistance effect among *PRX* genes might result from the multiple elements associated with different hormones and stresses [50]. In the present study, we found a large number of cis-elements implicated in hormone and stress responses (Figure 7 and Table S11), implying that the regulatory elements within *PRX* genes in different species are relatively conservative, which may be great contributors to the functional resilience of *LcPRX* genes. Among the 77 *LcPRX* genes, 73 *LcPRX* genes (except for *LcPRX18*/*28*/*32*/*75*) contained at least one of the four hormone response elements (gibberellin, abscisic acid, methyl jasmonic, and salicylic acid); 42 *LcPRX* genes (*LcPRX1*/*2*/*3*/*4*/*5*/*6*/*9*/*10*/*17*/*19*/*20*/*21*/*23*/*25*/*27*/*28*/*29*/*30*/*32*/*33*/*34*/*36*/*38*/*41*/*42*/*44*/*48*/*52*/*54*/*57*/*58*/*59*/*60*/*62*/*63*/*67*/*68*/*69*/*72*/*74*/*75*/*76*) contained various numbers of drought elements (MBS); 20 *LcPRX* genes (*LcPRX1*/*3*/*5*/*6*/*8*/*27*/*30*/*31*/*33*/*34*/*35*/*37*/*39*/*44*/*45*/*50*/*52*/*60*/*76*/*77*) contained low-temperature elements (LTR) with different quantities. These data suggest that *LcPRX* genes may be broadly involved in complex signaling pathway regulation and in responses to environmental stresses through a complex mechanism.

It is well known that gene expression patterns can provide important clues about gene function [34]. In this study, all of the 77 *LcPRX* genes, except for 6 (*LcPRX11*/*18*/*23*/*25*/*64*/*70*) with expression in only one examined tissue, had distinct tissue-specific expression in the different tissues (Figure 8 and Table S12), showing the functional dissimilation of *LcPRX* genes. This is consistent with the results of phylogenetic relationships and gene structure analyses. In addition, we have already seen most genes in subfamily IV-a, IV-b, and IV-d genes expressed in almost all tissues, suggesting that they might play basic and important roles for the litchi. Notably, the largest number of *LcPRX* genes with high expression levels were found in roots. Similar observations were also reported in Arabidopsis [29] and maize [28], proving that *PRX* genes may be critical for root function in plants. Lastly, 21 *LcPRX* genes (*LcPRX5*/*16*/*17*/*21*/*29*/*38*/*40*/*41*/*46*/*49*/*50*/*52*/*53*/*55*/*58*/*59*/*60*/*63*/*66*/*67*/*71*) had predominant expression in male flowers, female flowers, and ovaries. Similar phenomena were observed in foxtail millet [51], implying that *PRX* genes may play important roles in the differentiation and development of flower organs. In conclusion, the results provide a basis for the functional exploration of *LcPRX* gene family members.

An increasing body of research points to the fact that CIII PRXs are closely related to the resistance of plants, including cold resistance, drought resistance, salt resistance, etc., and they are important protective enzymes in plants [63,64]. This study provided a conclusion that many cis-acting elements related to hormones and stresses were predicted in the promoter regions of *LcPRX* genes (Figure 7 and Table S11). To further explore the possibility of the *LcPRX* genes responding to diverse stresses (cold, heat, drought, and salt), we selected 42 genes for qRT-PCR analysis based on the results of cis-acting element and heatmap analysis. The results suggested that the majority of the *LcPRX* genes were induced, and only a few of them were slightly induced by at least one of the four stress treatments (Figure 9). Similar results were also found for the *PRX* genes in grapevine (*Vitis vinifera*) [13] and tobacco [50]; for instance, 30 *VvPRX* genes were induced by drought and NaCl treatments; more than half of *NtPRX* genes (105) were induced by cold, NaCl, and drought treatments. Significantly, among abiotic stress-induced *LcPRX* genes, some were rapidly and highly expressed at 3 h after treatments, suggesting that these genes may be important pioneering in the process of litchi resistance to abiotic stresses; by contrast, the expression level of some gradually increased at 6 h or 12 h after treatments, showing that these genes were involved in the late reactions of abiotic stresses; additionally, the expression of others reached the highest levels with the extension of time, implying that these genes would potentially continue to function. Interestingly, a few *LcPRX* genes were lowly expressed after at least one of the four stress treatments. We speculate that these genes may also have defensive and other specific functions in litchi. Remarkably, six pairs of SD genes (*LcPRX17*/*21*, *LcPRX49*/*29*, *LcPRX50*/*35*, *LcPRX67*/*75*, *LcPRX74*/*16*, and *LcPRX74*/*22*) showed similar expression levels at four time points after stress treatments. Although their expression levels peaked at different time points, these SD genes showed similar trends throughout the adversity resistance reaction process of litchi. Together, these results demonstrate that *LcPRX* genes respond to multiple abiotic stresses, which will be candidate targets for the genetic improvement of litchi. The findings will significantly contribute to breeding excellent varieties of litchi. However, the intricate regulatory mechanisms of stress resistance in relation to these *PRX* genes in litchi still necessitate further elucidation. Thus, further investigations into individual *LcPRX* genes are warranted in the future. Revealing the specific functions of these individual *LcPRX* genes will deepen our understanding of the molecular mechanisms underlying stress resistance in litchi and provide a reference for establishing abiotic resistant litchi cultivars.

## 4. Materials and Methods

### 4.1. Plant Materials and Stress Treatments

One-year-old litchi seedlings of an early maturity variety, Feizixiao (the widest planting areas and the most mature cultivation technology in China) [2] located at the Institute of Fruit Tree Research, Guangdong Academy of Agricultural Sciences, Guangzhou, China (113°22′41.200″ E, 23°9′32.418″ N), were selected in this study. The seedling soil was composed of 60% sandy red soil, 20% peat soil, and 20% coconut bran silk, with pH values between 5.5 and 6.5, suitable for litchi seedling growth. Subsequently, the seedlings were routinely managed in a greenhouse, and we selected sixty seedlings at the same developmental stage (with twenty-five leaves) to carry out experiments in April 2023. To investigate the abiotic stress responses of litchi, cold treatment, heat treatment, drought treatment, and salt treatment (each treatment group consisting of fifteen seedlings) were treated with 4.0 ± 1.0 °C, 38.0 ± 0.5 °C, 20% (*w*/*v*) PEG6000, and 400 mmol/L sodium chloride (NaCl), respectively, according to the growth conditions of litchi in complex and variable environments [6]. Their leaves were collected at 0, 3, 6, 12, and 24 h intervals after treatments. The upper leaves of three seedlings per time point were mixed together as one biological replicate, with three biological replicates used for each sample. All the materials were immediately frozen in liquid nitrogen and kept at −80 °C until RNA extraction. 

### 4.2. Screening and Domain Identification of LcPRX Genes

We acquired the Hidden Markov Model (HMM) profile of the PRX domain (PF00141) from the protein family (Pfam) database (https://www.ebi.ac.uk/, accessed on 22 July 2023). Based on the HMM profile, we then used the HMMER 3.0 software to search *PRX* genes from the litchi genome database [1], with the expected value (E-value) cutoff set to 1 × 10^−5^. Subsequently, all candidate LcPRX protein sequences were further identified by the CDD database (https://www.ncbi.nlm.nih.gov/cdd/?term=, accessed on 22 July 2023), the InterPro database (https://www.ebi.ac.uk/interpro/search/sequence/, accessed on 22 July 2023), and the SMART database (http://smart.embl-heidelberg.de/, accessed on 22 July 2023). Sequences without a PRX domain were removed. A total of 77 *LcPRX* genes were finally obtained and named according to their positional order on fifteen chromosomes.

### 4.3. Basic Physicochemical Properties of LcPRX Genes

The protein length, molecular weight, theoretical isoelectric point (pI), instability index, aliphatic index, and grand average of hydropathicity (GRAVY) were calculated using the ExPASy online tool (https://web.expasy.org/protparam/, accessed on 28 July 2023). For the number of transmembrane domains, the TMHMM 2.0 online tool (https://services.healthtech.dtu.dk/services/TMHMM-2.0/, accessed on 6 August 2023) was employed, while the subcellular location of each LcPRX protein was predicted using the PSORT online tool (https://www.genscript.com/psort.html, accessed on 10 August 2023).

### 4.4. Phylogenetic, Gene Structure, and Conserved Motif Analysis of LcPRX Genes

The generation of an unrooted maximum likelihood (ML) phylogenetic tree for PRX protein sequences in litchi and Arabidopsis (Table S1), along with bootstrap testing replicated 1000 times, was performed using MEGA 7.0 software. The gene structures of *LcPRX* genes were aligned using GSDS 2.0 (http://gsds.gao-lab.org/, accessed on 20 August 2023). The conserved motifs of the *LcPRX* genes were sought using the MEME online tool (https://meme-suite.org/meme/tools/meme, accessed on 28 August 2023) and visualized using TBtools-II v2.0 software [65]. Then, the conserved motifs were identified as to whether or not there was a PRX domain using the CDD database.

### 4.5. Chromosomal Distribution and Colinear Analysis of LcPRX Genes

The information on location for each *LcPRX* gene was obtained from the genome annotation of litchi. The chromosomal positions of *LcPRX* genes were visualized using TBtools-II v2.0. The One Step MCScanX from TBtools-II v2.0 was used to identify the duplications with default parameters and was used to analyze *PRX* genes in litchi vs. itself and litchi vs. Arabidopsis/rice, respectively. Then, the results were visualized using the Advanced Circos plugin from TBtools-II v2.0. The rates of nonsynonymous substitution (Ka) and synonymous substitution (Ks) were calculated using the Simple Ka/Ks Calculator (NG) plugin from TBtools-II v2.0. Ka/Ks rate > 1 indicates positive evolution, Ka/Ks rate = 1 indicates neutral evolution, and Ka/Ks rate < 1 indicates negative evolution.

### 4.6. Secondary Structure, Cis-Acting Element, and Transcriptome Analysis of LcPRX Genes

The secondary structures of the *LcPRX* genes were predicted using the SOPMA online tool (https://npsa-prabi.ibcp.fr/cgi-bin/npsa_automat.pl?page=npsa_sopma.html, accessed on 16 September 2023). The 2000 bp upstream sequences of coding DNA sequences (CDS) were analyzed for cis-acting elements using the PlantCARE online tool (http://bioinformatics.psb.ugent.be/webtools/plantcare/html/, accessed on 22 September 2023). Then, the results were organized and visualized using the HeatMap from TBtools-II v2.0. The transcriptome data, including seed, aril, root, leaf, male flower, female flower, ovary, carpopodium, and pericarp, were downloaded from the litchi genome database. Subsequently, the expression levels of *LcPRX* genes in different tissues were normalized according to FPKM (Fragments Per Kilobase of transcript per Million mapped reads) and drawn using TBtools-II v2.0.

### 4.7. qRT-PCR Analysis of LcPRX Genes

Total RNA extraction was performed using the Plant Total RNA kit with the DNase I enzyme (SIMGEN, Hangzhou, China), and cDNA synthesis was carried out using the cDNA First Strand Synthesis kit (SIMGEN, Hangzhou, China). The qRT-PCR analysis was conducted using the 2 × SYBR Green PCR Mix kit (SIMGEN, Hangzhou, China) and the QuantStudio^TM^ 3 Real-Time PCR system (Thermo Fisher Scientific, Guangzhou, China). Relative expressions were calculated using the 2^−∆∆CT^ method and visualized using the SigmaPlot 14.0 software. The significances (*p* < 0.05) were calculated using the DPS 9.01 software with LSD multiple tests. Three technical replicates were applied for each gene. Primers for *LcPRX* genes and the *Actin* gene [66] are detailed in Table S2.

## 5. Conclusions

This study successfully identified 77 *LcPRX* genes and mapped them onto fifteen chromosomes in litchi. According to phylogenetic relationships, these *LcPRX* genes were classified into eight subfamilies (I, II-a, II-b, III, IV-a, IV-b, IV-c, and IV-d). The subcellular localization of the *LcPRX* genes might indicate that these genes mainly play a certain role in chloroplasts. The structural diversity of the *LcPRX* genes suggested their functional diversity. The cis-acting elements analysis of the *LcPRX* genes proved that they were widely involved in the growth, hormonal, and stress responses of litchi. The expression patterns of the *LcPRX* genes showed that they were expressed distinctly in different tissues of litchi. Furthermore, it is important to thoroughly investigate the regulatory functions of the *LcPRX* genes, especially their roles in resistance to abiotic stresses. These comprehensive findings shed light on the molecular basis of the *LcPRX* gene family and will lay the foundation for future studies on verifying the precise regulation of *LcPRX* genes.

## Data Availability

The data are contained within the present article and Supplementary Materials.

## Supplementary Materials

The following supporting information can be downloaded at: https://www.mdpi.com/article/10.3390/ijms25115804/s1.
