# Correction: Yang et al. Genome-Wide Identification and Expression Analysis of the Class III Peroxidase Gene Family under Abiotic Stresses in Litchi (*Litchi chinensis* Sonn.). *Int. J. Mol. Sci.* 2024, *25*, 5804

**DOI:** 10.3390/ijms251910249

**Published:** 2024-09-24

**Authors:** Jie Yang, Rong Chen, Xu Xiang, Wei Liu, Chao Fan

**Affiliations:** Guangdong Provincial Key Laboratory of Tropical and Subtropical Fruit Tree Research, Key Laboratory of South Subtropical Fruit Biology and Genetic Resource Utilization, Ministry of Agriculture and Rural Affairs, Institute of Fruit Tree Research, Guangdong Academy of Agricultural Sciences, Guangzhou 510640, China; yangjie95222@126.com (J.Y.); rongchen9821@126.com (R.C.); xiangxu@vip.163.com (X.X.); liuwei1@gdaas.cn (W.L.)

In the original publication [1], there was a mistake in Ref. [66] “Wei, Y.Z.; Hu, F.C.; Hu, G.B.; Li, X.J.; Huang, X.M.; Wang, H.C. Differential expression of anthocyanin biosynthetic genes in relation to anthocyanin accumulation in the pericarp of *Litchi Chinensis* Sonn. *PLoS ONE* **2011**, *6*, e19455” as published. The reference was incorrect due to our mistake. The corrected Ref. [66] appears below.

66. Sun, J.H.; Cao, L.L.; Li, H.L.; Wang, G.; Wang, S.J.; Li, F.; Zou, X.X.; Wang, J.B. Early responses given distinct tactics to infection of *Peronophythora litchi* in susceptible and resistant litchi cultivar. *Sci. Rep*. **2019**, *9*, 2810.

With this correction, the order of other references does not need to be adjusted. The authors state that the scientific conclusions are unaffected. This correction was approved by the Academic Editor. The original publication has also been updated.
